# Effect of resistance training and chicken meat on muscle strength and mass and the gut microbiome of older women: A randomized controlled trial

**DOI:** 10.14814/phy2.16100

**Published:** 2024-06-18

**Authors:** Masataka Uchida, Jonguk Park, Shumpei Fujie, Koji Hosomi, Naoki Horii, Kohei Watanabe, Kiyoshi Sanada, Yasushi Shinohara, Kenji Mizuguchi, Jun Kunisawa, Motoyuki Iemitsu, Motohiko Miyachi

**Affiliations:** ^1^ Faculty of Sport and Health Science Ritsumeikan University Kusatsu Japan; ^2^ Artificial Intelligence Center for Health and Biomedical Research National Institutes of Biomedical Innovation, Health and Nutrition Osaka Japan; ^3^ Laboratory of Vaccine Materials and Laboratory of Gut Environmental System, Microbial Research Center for Health and Medicine National Institutes of Biomedical Innovation, Health and Nutrition Osaka Japan; ^4^ Laboratory of Neuromuscular Biomechanics, School of Health and Sport Sciences Chukyo University Toyota Japan; ^5^ Institute for Protein Research, Osaka University Osaka Japan; ^6^ Faculty of Sport Sciences Waseda University Saitama Japan

**Keywords:** aging, chronic resistance exercise, gut bacteria, muscle mass, muscle strength

## Abstract

This study investigated the effects of white meat, such as chicken, intake combined with resistance training on muscle mass and strength in the elderly women, and whether the underlying mechanism involves changes in the gut microbiota. Ninety‐three volunteers (age 59–79 years) were randomly allocated to sedentary control with placebo (Sed + PL) or chicken meat (Sed + HP) and resistance training with placebo (RT + PL) or chicken meat (RT + HP). Resistance training sessions were performed 3 d/week for 12 weeks using leg extensions and curls. Boiled chicken meat (110 g, containing 22.5 g protein) was ingested 3 d/week for 12 weeks. Maximal muscle strength and whole‐body lean mass increased significantly in the RT + PL group compared to the Sed + HP group, and the RT + HP group showed a significantly greater increase than the Sed + HP and RT + PL groups. Additionally, the gut microbiota composition did not change before or after the interventions in any of the four groups. Moreover, the individual comparison of gut bacteria using false discovery rate‐based statistical analysis showed no alterations before or after the interventions in the four groups. Resistance training combined with chicken meat intake may effective have increased muscle mass and strength without drastically modifying the gut microbiota composition in elderly women.

## 1 INTRODUCTION

Muscle mass and strength decline with age. Many aging‐associated factors contribute to decreased muscle mass and strength, including imbalance in muscle protein synthesis and degradation, low physical activity, low habitual protein intake, and attenuation of hormone secretion. Aging‐induced loss of muscle mass and strength leads to the developing sarcopenia (Rosenberg, 1997). Sarcopenia is associated with physical disabilities (Baumgartner et al., 1998) and an increased risk of falls, fractures (Walsh et al., 2006), and mortality (Sanada et al., 2018), and it may lead to diabetes (Karakelides & Nair, 2005), cardiovascular diseases (Sanada et al., 2010), and dementia (Sanada et al., 2010). Further studies are necessary to prevent age‐related loss of muscle mass and strength. In postmenopausal women, loss of skeletal muscle mass and function is accelerated because of decreasing sex steroid hormone secretion (Messier et al., 2011). Moreover, the hypertrophic response to resistance training decreases in elderly women compared with that in elderly men (Bamman et al., 2003).

Habitual resistance exercise is an effective method for preventing age‐related loss of muscle mass and strength. Resistance exercise promotes muscle protein synthesis, resulting in augmentation of muscle mass and strength (Sato et al., 2014; Watanabe, Tanimoto, et al., 2015). Therefore, adequate daily protein intake is necessary to increase muscle mass via resistance exercise‐induced muscle protein synthesis. The recommended dietary allowance for protein intake in the elderly is 1.2 g/kg body weight (BW)/day (Morton et al., 2018). Furthermore, a recent study on adults showed that an increase in total protein intake (1.6 g/kg BW/day) combined with resistance training may promote increased lean body mass (LBM) (Morton et al., 2018). Meat, fish, soybeans, and milk are protein‐rich sources. Previous studies have associated red meat consumption with increased mortality due to cardiovascular disease (Schmid, 2011), cancer (Bouvard et al., 2015), and risk of type II diabetes (Pan et al., 2011). The intake of white meat, such as chicken, instead of red meat, has been shown to be effective (Bernstein et al., 2010; Willett & Stampfer, 2013). However, the effects of chicken meat intake combined with resistance training on muscle mass and strength in the elderly and the underlying mechanisms are unclear.

Several studies have revealed the effects of the gut microbiota on muscle mass and strength (Turnbaugh et al., 2006; Yang et al., 2017). In a cross‐sectional study on healthy adults and elderly with sarcopenia, the α‐diversity of the gut microbiota reduced with loss of muscle mass and strength (Kang et al., 2021). Additionally, a study reported a positive correlation between the *Ruminococcus genus* and grip strength (Kang et al., 2021). Hence, the aging‐induced loss of muscle mass and strength may be related to changes in the diversity and/or composition of the gut microbiota (Kang et al., 2021). In a recent study, 12‐week resistance training in rats altered the β‐diversity and composition of the gut microbiota (Castro et al., 2021). Furthermore, a study showed that chronic meat consumption in young adults increased the diversity and altered the gut microbiota composition (Shi et al., 2021). However, the effects of the gut microbiota on increased muscle mass and strength in the elderly due to protein intake, such as white meat, combined with resistance training have not been studied, and elucidating these effects may help propose an effective approach to improve muscle mass and strength in the elderly.

Therefore, in this study, we aimed to investigate the effects of resistance training and chicken meat intake, which increase muscle mass and strength, and their combination on the gut microbiota in elderly women. We hypothesized that the combination of resistance training and chicken meat intake in elderly women alters the gut microbiota, which further affects the increased muscle mass and strength. To test this hypothesis, we conducted a randomized controlled intervention trial to examine the effects of 12 weeks of high‐intensity resistance exercise combined with chicken meat intake on muscle mass, strength, gut microbiota, and the association among these changing parameters.

## 2 MATERIALS AND METHODS

### 2.1 Ethical approval

All the participants voluntarily provided written informed consent before participating in the study. This study was approved by the Ethics Committee of Ritsumeikan University (BKC‐2018‐060) and was conducted in accordance with the Declaration of Helsinki. The study was registered in the University Hospital Medical Information Network Clinical Trials Registry (UMIN000038253).

### 2.2 Participants

Community‐dwelling elderly women were recruited from Japan. In total, 103 participants were recruited through advertisements published in the local press and posters at community health and recreation centers. Of the 103 participants, 10 individuals who were performing habitual exercise had a history of cardiovascular disease, severe liver disease, chronic renal failure, gynecological disease, joint disorder, or mental disorder; therefore, they were excluded from the study. Lastly, 93 healthy older sedentary elderly women were included in this study.

### 2.3 Study design

After obtaining the informed consent, group allocation was performed using computer‐generated random numbers. Through a randomized controlled intervention trial, the participants were randomly divided into the following four groups: the sedentary control and placebo intake (Sed + PL) group, the sedentary control and chicken meat intake (Sed + HP) group, the resistance training and placebo intake (RT + PL) group, and the resistance training and chicken meat intake (RT + HP) group. The researchers who assessed each parameter were blinded to the group allocations. Body composition, muscle mass, muscle strength, physical activity, energy intake, and gut microbiota were assessed before and after the 12‐week resistance training with chicken meat intake. The fecal samples and fasting blood samples were collected at least 48 h after the last resistance exercise session to avoid the influence of acute exercise effects. All the participants were instructed not to perform strenuous exercise or drink alcohol for at least 24 h before fecal sampling. All participants were instructed not to eat or drink fluids, except water, for at least 10 h before reporting to the laboratory. Serum and plasma samples were centrifuged at 1500 × *g*, 15 min, at 4°C immediately after collection and stored at −80°C. The experimental room temperature was maintained at 24 ± 1°C during measurements. All the participants were instructed to maintain their physical activity and diet during the interventions, excluding chicken meat and/or placebo intake and/or resistance training.

### 2.4 Chicken meat and/or placebo intake

In the Sed + HP and RT + HP groups, steamed chicken breast meat containing an average calorie content of 164 kcal and an average protein content of 22.5 g per 110 g serving was ingested thrice a week for 12 weeks. The Sed + PL and RT + PL groups ingested a protein‐free carbohydrate control food diet with a similar energy content as the high‐protein diet. The RT + PL and RT + HP groups ingested their respective servings within 30 min of resistance exercise, whereas the Sed + HP and Sed + PL groups took a placebo between meals. If the rate of participation in the 36 chicken meat intakes was ≤90%, the individual was excluded from statistical analysis because of “low adherence to nutrition.”

### 2.5 High‐intensity resistance training

The RT + PL and RT + HP groups performed three sets of 10 repetitions of leg extensions and leg curls at an intensity of 70% of the one‐repetition maximum (1RM) 3 d/week on nonconsecutive days for 12 weeks (Watanabe, Sato, et al., 2015). The rest period between the sets was 2 min. Before the resistance exercise, a 3‐min walk on a treadmill and a 5‐min stretch of the lower limbs were performed for warm‐up, and a 5‐min stretch was performed for cooldown after the resistance exercise. In the 1RM measurement for setting the training intensity, the weight load of each participant was gradually increased, and the exercises were repeated until the participant was unable to lift the weight, at which point the 1RM was measured. The 1RM was assessed once every 2 weeks during the intervention period. If the rate of participation in the 36 resistance training sessions was ≤90%, the individual was excluded from the statistical analysis because of “low adherence to training.”

### 2.6 Measurements

#### 2.6.1 Anthropometric measures

BW was measured using a body composition analyzer (RD‐801, TANITA, Tokyo, Japan). Height was measured by height meter (WB‐510, TANITA, Tokyo, Japan). Those measured to the nearest 0.1 kg and 0.1 cm, respectively. Body mass index (BMI; kg/m^2^) was calculated using BW and height.

#### 2.6.2 Body composition, muscle mass, and strength

Body composition was determined through dual‐energy X‐ray absorptiometry (Lunar Prodigy, GE Healthcare, Little Chalfont, UK). Fat mass and % body fat of the total body mass, and the LBM of arms, trunk, and legs were measured as previously described (Watanabe, Sato, et al., 2015).

The maximum knee extension muscle strength was evaluated as an index of muscle strength. The participants were in a sitting position with their right leg fixed to a custom‐msade dynamometer (Takei Scientific Instruments Co., Ltd., Niigata, Japan) equipped with a force transducer (LU‐100KSE; Kyowa Electronic Instruments, Tokyo, Japan) and their hip and knee joints fixed at 90°, as previously described (Watanabe et al., 2020). They were instructed to gradually increase the knee extension force from the baseline to the maximum for over 2–3 s and then maintain it at the maximum force for 2 s. The maximum of the two measurements was used as the representative value. The participants performed triple‐leg extension with submaximal contraction at a low workload 5 min before the measurement for warm‐up and familiarization.

#### 2.6.3 Physical activity, energy intake, and nutritional status

Physical activity was evaluated using a triaxial accelerometer (SHINE2; MISFIT, Kyoto, Japan). The accelerometer was attached to the left wrist of each participant. Measurements were recorded for 2 weeks before the intervention and before the end of the intervention, and the average amount of physical activity for consecutive 7 days was used as the amount of physical activity/day. Dietary energy intake and nutritional status were assessed using a validated brief questionnaire containing 73 items based on a self‐administered diet history before the intervention and before the end of the intervention, as previously described (Kobayashi et al., 2011).

#### 2.6.4 Gut microbiota

The fecal samples were collected using the guanidine thiocyanate solution in collection tubes (TechnoSuruga Laboratory Co., Ltd, Shizuoka, Japan) for analyzing the fecal microbiota (Hosomi et al., 2017). After collection, the samples were immediately stored at 4°C until DNA extraction. The DNA samples were subjected to 16S ribosomal RNA (rRNA) metagenomic analysis using next‐generation sequencing (NGS).

According to previous study, we extracted fecal DNA using the bead‐beating method and an automatic nucleic acid extraction system (Gene Prep Star PI‐80X; Kurabo Industries, Ltd., Osaka, Japan) and subjected to NGS of the 16S rRNA gene amplicon, as previously described (Hosomi et al., 2017). Sequencing of the 16S rRNA gene amplicon in fecal DNA was based on previous study (Hosomi et al., 2017). The V3–V4 regions of the bacterial 16S rRNA were amplified using the following primers: forward, 5′‐ TCGTCGGCAGCGTCAGATGTGTATAAGCGACAGCCTACGGGNGGCWGCAG‐3′ and reverse, 5′‐GTCTCGTGGGCTCGGAGATGTGTATAAGAGACAGGACTACHVGGGTATCTAATCC‐3′ (Klindworth et al., 2013). A DNA library was prepared using the Nextera kit Set A (Illumina, San Diego, California, USA, FC‐131‐2001). Barcoded amplicons were paired‐end sequenced using the MiSeq system with MiSeq Reagent Kit version 3 (600 cycles) chemistry (Illumina, San Diego, California, USA, MS‐102‐3003). The sequencing data were analyzed using the Quantitative Insights into Microbial Ecology (QIIME) software package (Caporaso et al., 2010) and QIIME Analysis Automating Script (Auto‐q) (10.5281/zenodo.1439555), as previously described (Mohsen et al., 2019). Primer sequences for the paired‐end sequencing reads were trimmed using Cutadapt Ver. 1.18 with the default settings. Open‐reference operational taxonomic unit (OTU) selections and taxonomy classifications were performed based on sequence similarity (>97%) using the UCLUST software (Edgar, 2010) and SILVA v128 reference sequence (Quast et al., 2013).

#### 2.6.5 Blood sampling

Fasting total cholesterol (Total‐Cho), high‐density lipoprotein cholesterol (HDL), triglycerides (TG), and glucose (FBG) were measured using standard enzymatic techniques based on previous studies (Watanabe, Sato, et al., 2015).

#### 2.6.6 Statistical analyses

All values were expressed as mean ± standard deviation (SD). Statistical evaluations for each parameter were performed using a two‐way analysis of variance (ANOVA) for repeated measures (time × group). One‐way ANOVA was used to compare the differences in changes from baseline to 12 weeks among the four groups. A Fisher's post hoc comparison test was performed to correct for multiple comparisons when the analyses revealed significant differences. The *p*‐value of <0.05 was defined as statistically significant. All statistical analyses were performed using the StatView software (ver. 5.0; SAS Institute, Minato‐ku, Tokyo, Japan).

The α‐diversity indices were calculated using the *estimate_richness* function in the R package “phyloseq” (McMurdie & Holmes, 2013). The β‐diversity index was calculated according to the Bray–Curtis distance of the genus‐level data and was generated using the *vegdist* function in the R package “vegan” (Oksanen et al., 2008). Principal coordinate analysis (PCoA) was performed using the *dudi.pco* function in the R package “ade4” (Dray & Dufour, 2007), and the PCoA figure was created by using the R package “ggplot2.” The dominant bacteria from the phylum to genus level were defined as a mean bacterial composition distribution of at least 0.05%. The Kruskal–Wallis test was performed to compare the relative abundance of taxa before and after the intervention in each group. Wilcoxon rank sum test (*wilcox*. *test* function of the “stats” R package) was performed to compare the changes before and after intervention in the RT + PL, Sed + HP, and RT + HP groups with those in the Sed + PL group. *q* value < 0.05 was defined as statistically significant. The *p*‐values of the dominant bacteria were adjusted using the false discovery rate (FDR) method. Herein, the statistical analyses of the gut microbiota analyses were performed using R (version 3.5.0).

## 3 RESULTS

**FIGURE 1:**
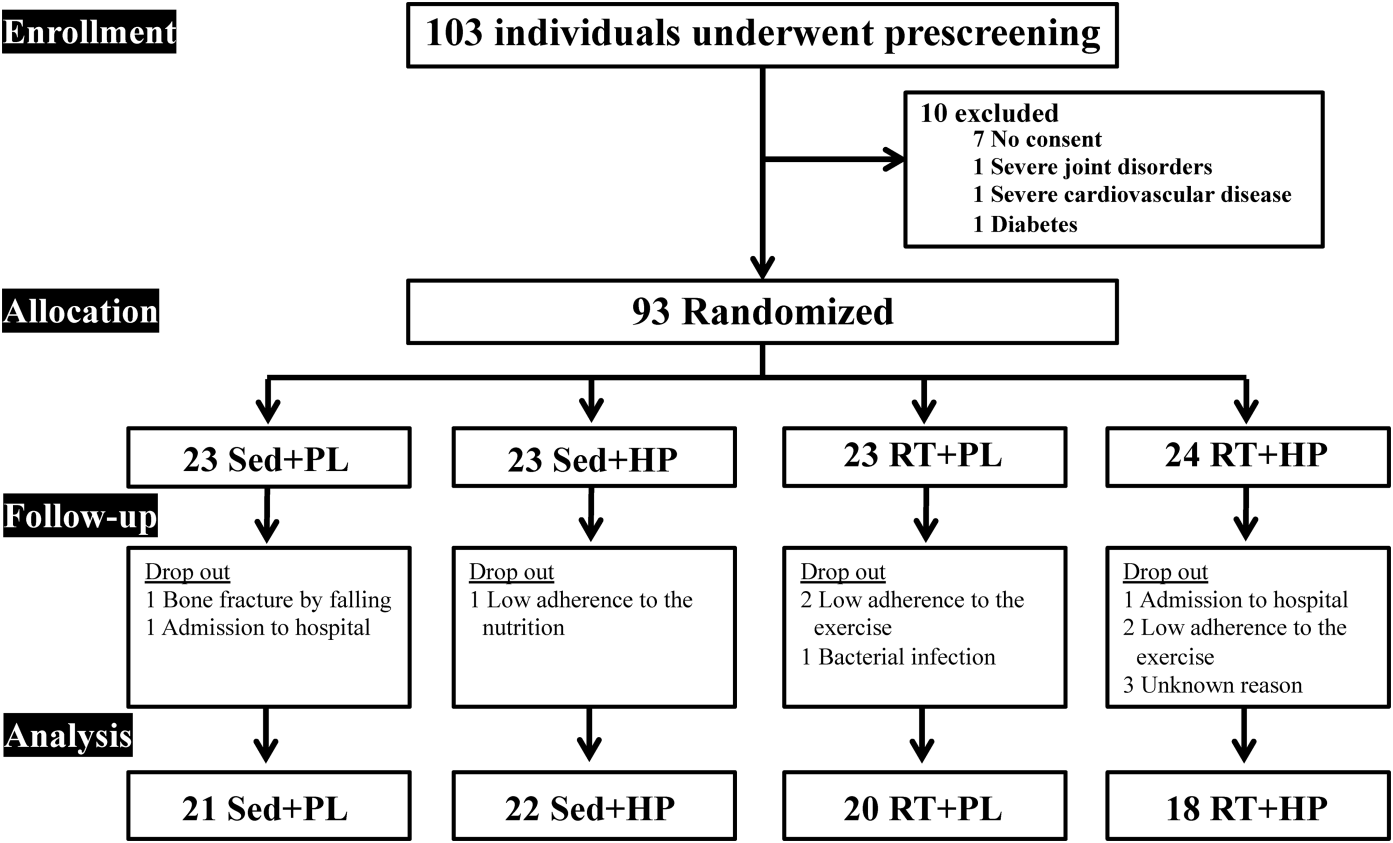
Flowchart showing the distribution of participants through the intervention.

The participant flowchart is presented in Figure 1. A total of 103 elderly women were screened and 93 participants were enrolled in this study. Of these, 12 participants were excluded from the study during follow‐up, and 81 completed the outcome measurements before and after the intervention (*n* = 21, Sed + PL group [91.3%]; *n* = 22, Sed + HP group [95.6%]; *n* = 20, RT + PL group [87.0%]; and *n* = 18, RT + HP group [78.3%]) (Figure 1).

### 3.1 Before intervention

No significant differences in age, height, BW, BMI, percentage of body fat, muscle mass, muscle strength, Total‐Cho, HDL, TG, FBG, or daily physical activity were observed among the four groups before the intervention (Table 1). However, the lower limb and whole LBM of the participants in the RT + PL group were significantly lower than those in the RT+ HP group.

**TABLE 1:** Participants characteristics, muscle mass, strength, blood parameters, and total calorie consumption before and after interventions.

	Sed + PL (*N* = 21)		Sed + HP (*N* = 22)		RT + PL (*N* = 20)		RT + HP (*N* = 18)	
	Before	After	Before	After	Before	After	Before	After
Body weight (kg)	54.3 ± 9.0	54.3 ± 9.5	54.7 ± 8.7	54.6 ± 8.9	57.0 ± 9.5	56.7 ± 9.5	49.7 ± 7.5	49.9 ± 7.2
BMI (kg/m^2^)	22.6 ± 3.0	22.6 ± 2.9	22.6 ± 3.6	22.6 ± 3.6	23.0 ± 3.2	22.8 ± 3.1	21.1 ± 3.0	21.2 ± 2.8
Body fat (%)	32.8 ± 8.0	33.4 ± 8.0	32.4 ± 7.4	31.9 ± 7.5	32.1 ± 8.2	32.1 ± 8.0	30.1 ± 6.9	29.7 ± 6.2
Upper‐limb LBM (kg)	15.2 ± 1.9	15.1 ± 1.9	15.4 ± 1.6	15.5 ± 1.7	15.8 ± 1.6	15.6 ± 1.5	14.4 ± 1.3	14.4 ± 1.4
Lower‐limb LBM (kg)	15.7 ± 2.0	15.7 ± 2.1	16.0 ± 1.9	16.2 ± 2.1	16.7 ± 1.5	16.9 ± 1.6	15.1 ± 1.6	15.5 ± 1.6
Whole LBM (kg)	34.1 ± 3.9	33.9 ± 4.0	34.6 ± 3.5	34.9 ± 3.8	35.9 ± 3.2	35.9 ± 3.2	32.6 ± 2.9	33.1 ± 3.0
MVC (Nm)	99.5 ± 52.3	127.9 ± 56.0	102.5 ± 68.3	127.5 ± 60.7	91.7 ± 53.1	168.9 ± 70.2	90.4 ± 39.7	166.0 ± 58.3
Total‐Cho (mg/dL)	244.4 ± 49.1	228.0 ± 36.7	252.2 ± 39.6	232.3 ± 38.3	242.9 ± 36.3	241.7 ± 34.4	251.4 ± 34.1	237.0 ± 29.2
HDL (mg/dL)	80.0 ± 23.8	75.0 ± 19.2	82.4 ± 19.8	79.0 ± 22.5	83.3 ± 19.2	78.4 ± 20.4	86.5 ± 13.0	84. 1 ± 13.7
TG (mg/dL)	103.0 ± 40.2	105.9 ± 52.1	93.0 ± 39.6	99.5 ± 58.7	101.6 ± 36.3	110.5 ± 51.1	91.3 ± 49.6	102.6 ± 83.9
FBG (mg/dL)	108.0 ± 28.3	101.5 ± 18.0	99.3 ± 9.9	97.5 ± 12.1	98.3 ± 13.0	98.9 ± 9.6	102.7 ± 15.1	95.5 ± 24.6
Total calorie consumption (kcal/day)	1821.4 ± 243.8	1758.4 ± 269.6	1827.8 ± 188.5	1890.3 ± 207.4	1880.7 ± 246.1	1890.3 ± 276.2	1800.6 ± 206.4	1818.3 ± 200.3

*Note*: The values are expressed as mean ± SD. Sed + PL: sedentary control with placebo, Sed + HP: sedentary control with chicken meat, RT + PL: resistance training with placebo, RT + HP: resistance training with chicken meat.

Abbreviations: BMI, body mass index; FBG, fasting blood glucose; HDL, high‐density lipoprotein cholesterol; LBM, lean body mass; MVC, maximum voluntary contraction; Total‐Cho, total cholesterol; TG, triglycerides.

Furthermore, no significant differences in daily intake of total energy intake or protein, fat, and carbohydrate intake, and total calorie consumption were observed among the four groups before the intervention (Table 2).

**TABLE 2:** Energy intake and macronutrient contributions before and after interventions.

	Sed + PL (*N* = 21)		Sed + HP (*N* = 22)		RT + PL (*N* = 20)		RT + HP (*N* = 18)	
	Before	After	Before	After	Before	After	Before	After
Dietary energy intake (kcal/day)	1798.6 ± 689.9	1699.9 ± 488.0	1810.3 ± 574.6	1845.5 ± 508.7	1720.7 ± 473.6	1819.4 ± 492.4	1664.3 ± 442.0	1859.9 ± 543.4
Protein intake (g/day)	79.5 ± 31.0	77.1 ± 32.7	77.5 ± 24.1	79.2 ± 24.0	76.8 ± 33.0	78.9 ± 24.7	75.7 ± 24.0	84.3 ± 29.3
Fat intake (g/day)	62.6 ± 26.7	64.0 ± 19.6	56.0 ± 17.1	65.3 ± 20.3	53.4 ± 20.1	60.1 ± 18.3	54.9 ± 17.5	66.8 ± 19.9
Carbohydrate intake (g/day)	222.9 ± 102.1	195.5 ± 57.5	230.7 ± 89.3	222.3 ± 75.1	221.1 ± 69.1	226.2 ± 73.8	206.7 ± 71.6	219.3 ± 80.6

*Note*: The values are expressed as mean ± SD. Sed + PL: sedentary control with placebo, Sed + HP: sedentary control with chicken meat, RT + PL: resistance training with placebo, RT + HP: resistance training with chicken meat.

### 3.2 Participants characteristics, muscle mass, muscle strength, and blood parameters before and after the intervention

No significant differences in BW, BMI, percentage of body fat, muscle mass, muscle strength, Total‐Cho, HDL, TG, FBG, or daily physical activity were observed among the four groups before and after the intervention (Table 1).

The change in LBM of the lower limb before and after the intervention was significantly higher in the RT + HP group than in the Sed + PL groups (*p* < 0.05, Table 3). The change in whole LBM was significantly higher in the Sed + HP group compared with that in the Sed + PL group and the RT + HP group compared with that in the RT + PL group (*p* < 0.05, Table 3). Additionally, the change in muscle strength was significantly higher in the RT + PL and RT + HP groups compared with that in the Sed + PL and Sed + HP groups (*p* < 0.05, Table 3).

**TABLE 3:** Changes in participants characteristics, muscle mass, muscle strength, blood parameters, and total calorie consumption before and after interventions.

	Sed + PL (*N* = 21)	Sed + HP (*N* = 22)	RT + PL (*N* = 20)	RT + HP (*N* = 18)
Body weight (kg)	−0.0 ± 1.5	−0.1 ± 1.4	−0.2 ± 1.4	0.2 ± 1.0
BMI (kg/m^2^)	−0.0 ± 0.7	−0.1 ± 0.5	−0.1 ± 0.6	0.1 ± 0.4
Body fat (%)	0.6 ± 1.2	−0.5 ± 1.5*	−0.1 ± 1.2	−0.3 ± 1.5*
Upper‐limb LBM (g)	−109.8 ± 288.6	97.7 ± 437.7	−188.9 ± 584.9	11.2 ± 457.0
Lower‐limb LBM (g)	−13.4 ± 403.2	218.3 ± 557.4	141.3 ± 446.2	396.6 ± 518.0*
Whole LBM (g)	−162.0 ± 507.5	322.5 ± 780.7*	−52.1 ± 828.8	443.9 ± 710.5* ^,^ ***
MVC (Nm)	28.4 ± 32.6	25.0 ± 39.2	77.2 ± 45.8* ^,^ **	75.6 ± 54.9* ^,^ **
Total‐Cho (mg/dL)	−16.5 ± 26.6	−19.9 ± 18.6	−1.3 ± 33.3	−14.4 ± 23.0
HDL (mg/dL)	−4.9 ± 13.1	−3.4 ± 8.0	−4.9 ± 10.3	−2.4 ± 9.8
TG (mg/dL)	2.8 ± 34.9	6.5 ± 33.7	8.9 ± 46.9	11.3 ± 49.6
FBG (mg/dL)	−6.5 ± 15.8	−1.8 ± 8.8	0.6 ± 7.2	−7.2 ± 19.4
Total calorie consumption (kcal/day)	−23.5 ± 68.8	−6.9 ± 68.5	12.1 ± 65.6	−7.9 ± 50.4

*Note*: The values are expressed as mean ± SD. Sed + PL: sedentary control with placebo, Sed + HP: sedentary control with chicken meat, RT + PL: resistance training with placebo, RT + HP: resistance training with chicken meat.

Abbreviation: BMI, body mass index; FBG, fasting blood glucose; HDL, high‐density lipoprotein cholesterol; LBM, lean body mass; MVC, maximum voluntary contraction; Total‐Cho, total cholesterol; TG, triglycerides.

* *p* < 0.05 versus Sed + PL.

** *p* < 0.05 versus Sed + HP.

*** *p* < 0.05 versus RT + PL.

### 3.3 Energy and macronutrient intakes before and after the intervention

No significant differences were observed in the daily intake and changes in the daily intake of total energy, protein, fat, and carbohydrate, and total calorie consumption among the four groups before and after the intervention (Tables 2 and 4, respectively).

**TABLE 4:** The changes in energy intake and macronutrient contributions before and after interventions.

	Sed + PL (*N* = 21)	Sed + HP (*N* = 22)	RT + PL (*N* = 20)	RT + HP (*N* = 18)
Dietary energy intake (kcal/day)	−98.7 ± 600.6	35.1 ± 406.2	98.6 ± 361.5	195.6 ± 325.5
Protein intake (g/day)	−2.5 ± 36.1	1.8 ± 18.7	2.1 ± 25.0	8.6 ± 17.9
Fat intake (g/day)	1.4 ± 24.7	9.3 ± 13.2	6.7 ± 16.0	11.8 ± 16.7
Carbohydrate intake (g/day)	−27.4 ± 75.7	−8.4 ± 70.7	5.2 ± 55.7	12.6 ± 41.5

*Note*: The values are expressed as mean ± SD. Sed + PL: sedentary control with placebo, Sed + HP: sedentary control with chicken meat, RT + PL: resistance training with placebo, RT + HP: resistance training with chicken meat.

### 3.4 Gut microbiota composition before and after the intervention

**FIGURE 2:**
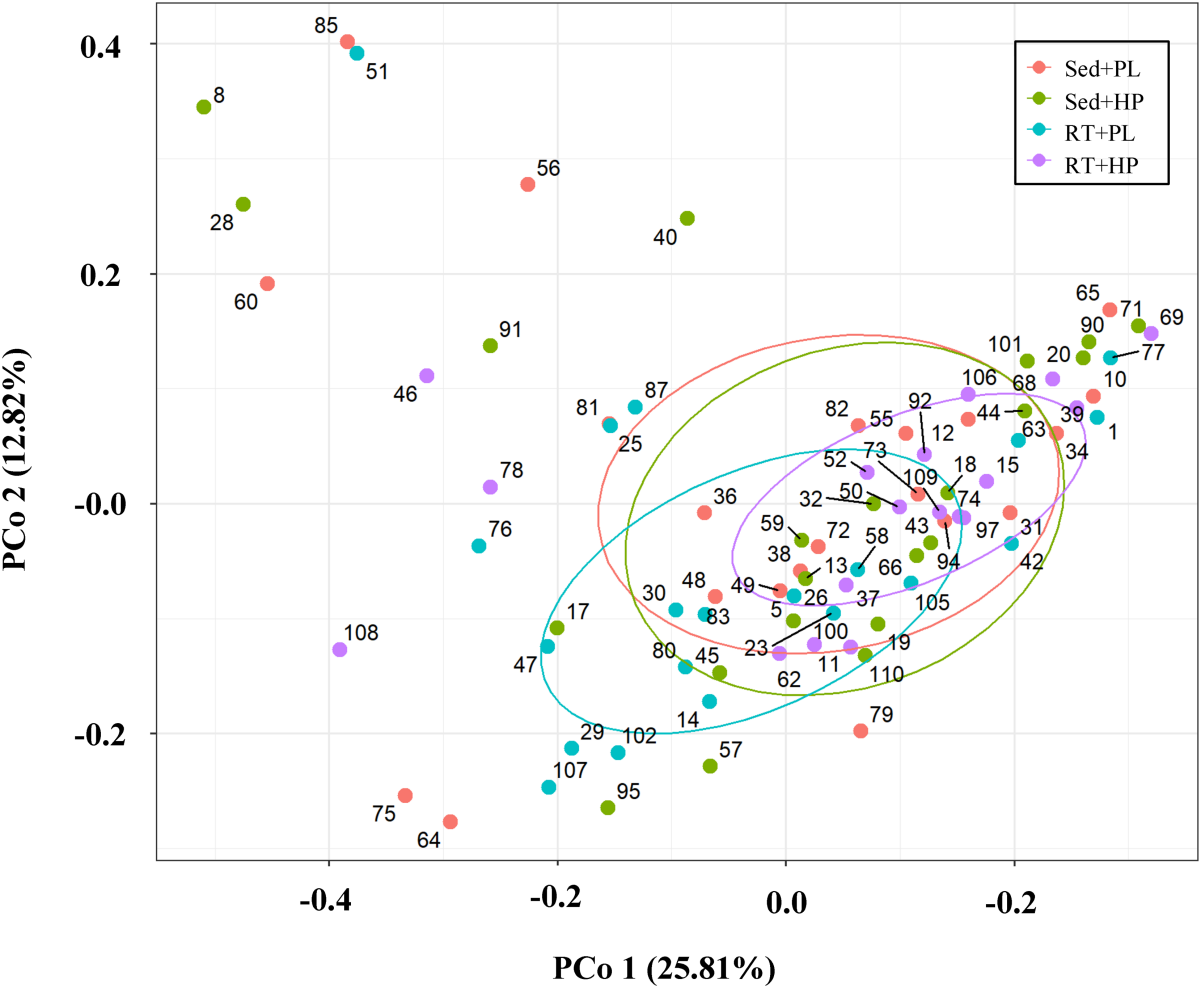
Principal coordinate analysis plots of weighted UniFrac distance metrics obtained from sequencing the 16S ribosomal RNA gene among the four groups at pre intervention. Axes represent the percentage of data explained by each coordinate dimension.

To confirm the bias in the gut microbiota composition before the intervention and the effects of each intervention, PCoA based on Bray–Curtis distance metrics was performed, and no significant differences in the gut microbiota composition were found before the intervention among the four groups (*r* ^2^ = 0.0306, *p* = 0.6843, Figure 2).

**FIGURE 3:**
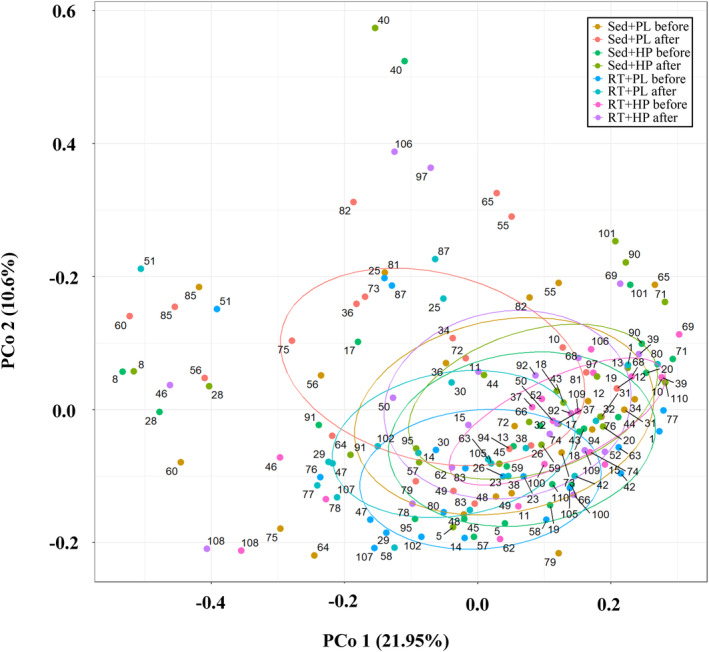
Principal coordinate analysis plots of weighted UniFrac distance metrics obtained from sequencing the 16S ribosomal RNA gene in all groups before and after the intervention. Axes represent the percentage of data explained by each coordinate dimension.

There was no significant interaction between the changes in the gut microbiota composition before and after the intervention among the four groups. However, significant differences were observed in the gut microbiota composition between each intervention (*r* ^*2*^ = 0.0334, *p* = 0.0009, Figure 3).

**FIGURE 4:**
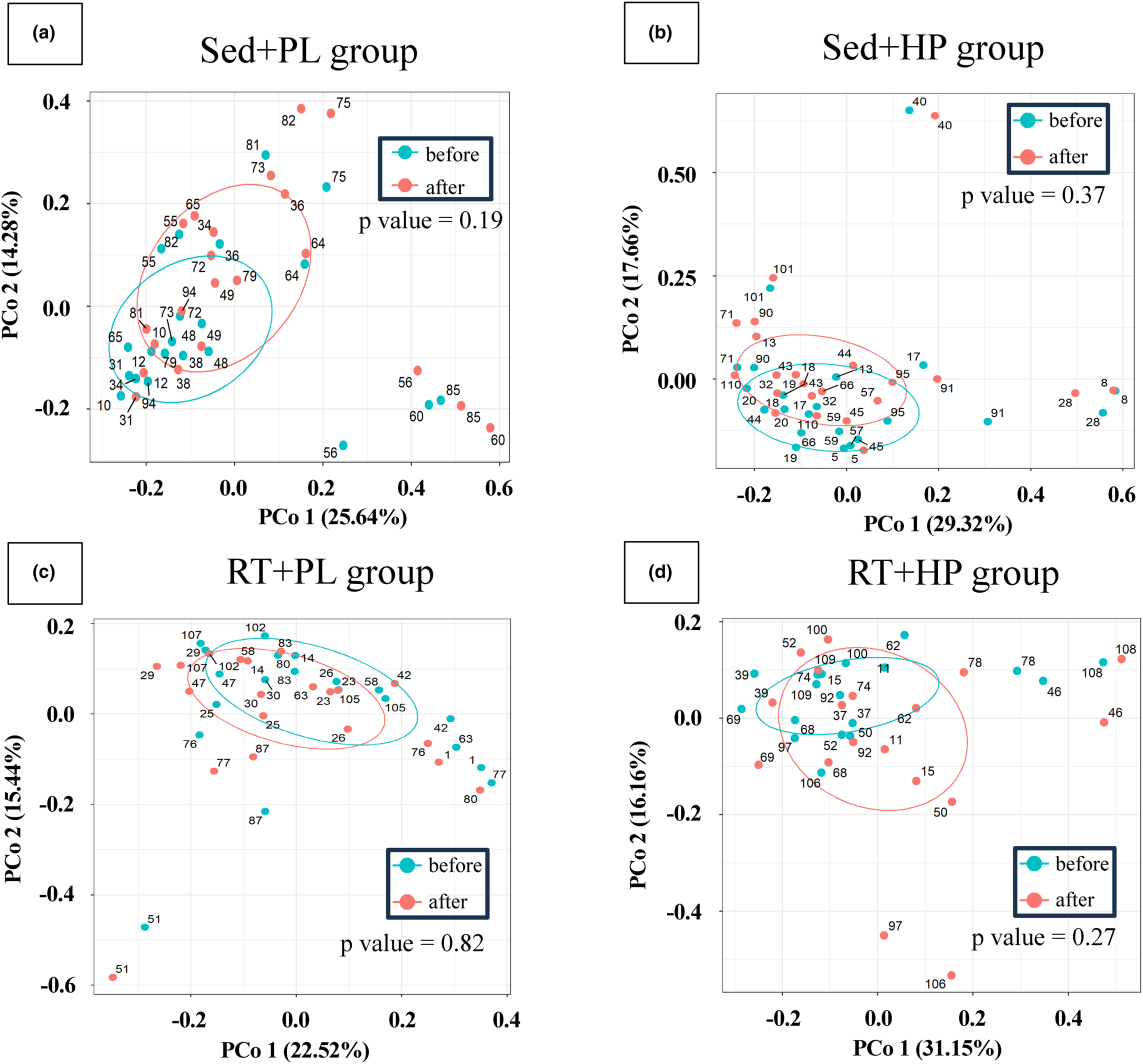
Principal coordinate analysis (PCoA) plots of weighted UniFrac distance metrics obtained from sequencing the 16S ribosomal RNA gene before and after the intervention in each group. PCoA plots of the sedentary control with placebo (a), sedentary control with chicken meat (b), resistance training with placebo (c), and resistance training with chicken meat (d) groups. Axes represent the percentage of data explained by each coordinate dimension.

PCoA analysis of the gut microbiota composition of each group revealed no significant difference regarding the effect of the intervention (Sed + PL: *r* ^2^ = 0.0182, *p* = 0.19; Sed + HP: *r* ^2^ = 0.0091, *p* = 0.37; RT + PL: *r* ^2^ = 0.0092, *p* = 0.82; and RT + HP: *r* ^2^ = 0.0328, *p* = 0.27; Figure 4).

### 3.5 Change in the gut bacteria before and after the intervention

Herein, we have comparatively analyzed the effects of intervention on the gut microbiota before, during, and after the intervention. The α‐diversity indices of the observed OTUs, Chao1, and Fisher in four groups were significantly higher before the intervention than those after the intervention, and those significant differences were also observed in FDR analysis (Table S1). Furthermore, significant differences in multiple gut bacteria were observed before and after the intervention among the four groups (Table S1). The changes before and after the intervention in the Sed + HP, RT + PL, and RT + HP groups were comparatively analyzed, using the gut bacteria with an abundance ratio of >1%, with the change in the Sed + PL group but no significant difference was observed in the α‐diversity index (Table S2). However, FDR analysis revealed no significant differences in multiple gut bacteria among the four groups before and after the intervention (Table S2). Additionally, statistical analysis based on the p value showed that the change in the *Eubacterium hallii group* genera composition before and after the intervention was lower in the RT + HP group compared with that in the Sed + PL group (Figure S1A).

### 3.6 Correlation between the gut bacteria and lower muscle mass

Change in *Eubacterium hallii group* genera composition before and after the intervention was significantly negatively correlated with the changes in the low LBM (Figure S1B, *r* = −0.323, *p* = 0.003).

## 4 DISCUSSION

This study aimed to investigate the effects of 12 weeks of high‐intensity resistance exercise combined with chicken meat intake on muscle mass, muscle strength, and gut microbiota in elderly women. Herein, high‐intensity resistance training intervention alone increased maximal muscle strength, whereas chronic chicken meat intake alone increased whole LBM. Interestingly, the combination of resistance training and chicken meat intake further increased maximal muscle strength and whole condition LBM compared with those by chicken meat intake or high‐intensity resistance exercise alone, suggesting the improved effects of the combined intervention on muscle mass and strength. Additionally, the gut microbiota composition did not change before and after these interventions in any of the four groups, and no significant differences were observed among the four groups. Moreover, individual examination of the gut bacteria using FDR‐based statistical analysis revealed no change before and after the interventions in the four groups. Thus, these results suggest that chronic chicken meat intake may have a combined effect with resistance training on muscle mass and strength, without drastically altering the gut microbiota composition in elderly women.

Red and white meats are rich in protein and very important sources of protein intake (Schmid, 2011). However, several epidemiological studies have shown that habitual meat intake, especially that of red meat, such as beef and pork, increases the incidence of cardiovascular disease and colorectal cancer (Bastide et al., 2015; Bujnowski et al., 2011; Pham et al., 2014). Furthermore, red meat, such as pork, intake may lead to increased microbial protein fermentation in the colon and associated changes in the gut microbiota (Zhang et al., 2020). Reportedly, red meat intake promotes dysbiosis of the gut microbiota and the production of metabolites that exert negative effects via the oxidation of trimethylamine (TMAO) metabolized by gut bacteria, indicating that high red meat intake negatively affects cardiovascular health (Buffa et al., 2022). In contrast, white meat (such as poultry) does not increase the metabolites to TMAO (Wang et al., 2019). Furthermore, the previous study showed that the differences in the gut microbiota composition were found among intake of chicken meat, red meat (beef and pork), and soy protein, and the protein intake from chicken meat increased the composition of beneficial Lactobacillus as compared with that from a soy protein diet and red meat diet in rats (Zhu et al., 2015). Moreover, the inflammatory response to the host was declined by the chicken meat intake as compared to soy protein intake in rats (Zhu et al., 2015). In a cross‐sectional study, a chicken‐fed population showed increased α‐diversity in the gut microbiota compared with that in a pork‐fed population (Shi et al., 2021). In this present study, the β‐diversity of the gut microbiota did not change dramatically by chicken intake alone or in combination with resistance training. Moreover, no difference in the change in the β‐diversity of the gut microbiota was found between each intervention in this present study. Additionally, FDR analysis revealed no significant changes in gut bacteria. Therefore, chronic chicken meat intake and resistance training did not drastically modify the gut microbiota composition in elderly women, and habitual intake of chicken meat may not negatively affect the gut microbiota compared with that of red meat.

A meta‐analysis of the combined effects of resistance exercise and protein intake in the elderly showed that the combined use of high‐protein diets was more effective in increasing LBM and muscle strength than resistance exercise alone (Liao et al., 2017). Moreover, a recent meta‐analysis showed that the most appropriate amount of total protein intake for maintaining and augmenting muscle strength with resistance training is 1.5 g/kg BW/day (Tagawa et al., 2022), and the protein intake for increasing or maintaining LBM ranges from 0.5 to 3.5 g/kg BW/day (Tagawa et al., 2020). Additionally, a previous study on elderly women showed that resistance exercise or red meat intake of 160 g/day (45 g protein) alone did not increase LBM, but their combination increased the LBM (Daly et al., 2014). In this study, the combination of chicken meat intake (22.5 g protein/110 g chicken meat) and resistance exercise increased muscle mass in the lower extremities of elderly women more than either of the individual interventions. The mean total protein intake in the combined group was 1.69 g/kg BW/day of total protein intake. Therefore, the combination of resistance exercise and chicken meat intake increased muscle strength, suggesting that the effects of chicken meat intake may be similar to those of other meat products.

Herein, the combination of resistance training and chicken meat intake increased muscle mass and strength without drastically modifying the gut microbiota composition in elderly women. However, *p*‐value‐based statistical analysis of individual gut bacteria revealed a decrease in the composition of *Eubacterium hallii group* genera when intervention included chicken meat intake and resistance training alone and the composition was significantly decreased with their combined intervention. A cross‐sectional study on professional rugby athletes, who performed habitual resistance exercise and had high protein intake, showed that the β‐diversity of the gut microbiota differed in nonathletes (Clarke et al., 2014). Furthermore, Clark et al. (2014). proposed that these adaptations in athletes may be caused by resistance exercise training combined with high‐protein diet intake over a long period, resulting in the modification of the gut microbiota in athletes. Therefore, in the case of short‐term (12‐week) exercise and nutritional interventions, as performed in this study, the net balance of muscle protein synthesis and degradation may affect muscle hypertrophy more intensely than the changes in the gut microbiota. Additionally, the gut microbiota of bodybuilders has shown a marked decrease in the *Eubacterium hallii group* genera composition compared with that in untrained adults (Jang et al., 2019). Interestingly, the *Eubacterium hallii group* genera composition has been positively correlated with blood C‐reactive protein levels in the elderly (Yu et al., 2018). Age‐related chronic inflammation may be involved in muscle loss by promoting protein degradation and inhibiting protein synthesis. Moreover, our previous study showed that resistance training attenuated inflammatory responses throughout systemic and various tissue levels in aged mice (Uchida et al., 2019). In this study, changes in the relative abundance of the *Eubacterium hallii group* genera were negatively correlated with changes in muscle mass. Hence, the *Eubacterium hallii group* genera composition decreased with resistance training may affect inflammatory responses in elderly individuals, resulting in increase in muscle mass.

This study had two limitations. First, in this study, the changes in lower limb LBM did not significantly change the resistance training or high‐protein diet intervention alone group as compared to the Sed + PL group, but the changes in lower limb LBM significantly increased in the combined intervention group as compared to the Sed + PL group. Therefore, we believed that no effect was observed with each intervention alone, and a significant increase in lower limb LBM was observed by the combined intervention. Second, in this study, changes in the relative abundance of the *Eubacterium hallii group* genera were negatively correlated with changes in muscle mass. The changes in the gut bacteria by the combined intervention are shown in this study. Therefore, further study needs to examine whether the gut microbiota is involved in the effect of the combined intervention.

To conclude, the combination of resistance training and steamed chicken meat intake may be effective in increasing muscle mass and strength without drastically modifying the gut microbiota composition in elderly women. Our findings indicate that chicken meat intake during the intervention did not cause meat‐induced dysbiosis (negative effects on the gut environment), as reported in previous studies, suggesting the positive effects of resistance training in the combined intervention.

## AUTHOR CONTRIBUTIONS

M.I., M.M., K.S., and M.U. conceived and designed research; M.I., M.U., K.H., N H., and M.I. performed experiments; M.U., J.P., N.H., and M.I. analyzed data; M.I., M.U., J.P., N.H., and M.M. interpreted results of experiments; M.I., M.U., J.P., and N.H. prepared figures; M.I., M.U., J.P., and M.M. drafted manuscript; M.I., M.M., K.S., M.U., J.P., N.H., S.F., K.H., K.W., Y.S., J.K., and K.M. edited and revised manuscript; and M.I., M.M., K.S, M.U., J.P., N.H., S.F., K.H., K.W., Y.S., J.K., and K.M. approved final version of manuscript.

## FUNDING INFORMATION

This work was supported by the Ito Foundation, Tokyo, Japan (#134, M. Iemitsu), Grants‐in‐Aid for Scientific Research from the Ministry of Education, Culture, Sports, Science, and Technology of Japan (KAKENHI: #22H03487 for M. Iemitsu; and #16 K00944 and #20H04117 for M. Miyachi), the Ministry of Health, Labour and Welfare (#201709002B for M. Miyachi), the Japan Agency for Medical Research and Development (AMED; 22ae0121035s0102 for K. Hosomi and 22ae0121042h0002 and 22ae0121035s0102 for J. Kunisawa), the Ministry of Health and Welfare of Japan and Public /Private R&D Investment Strategic Expansion PrograM (PRISM; 20 AC5004 for J. Kunisawa), and Programs for Bridging the gap between R&D and the IDeal society (society 5.0), and Generating Economic and social value: BRiDGE (J. Kunisawa).

## CONFLICT OF INTEREST STATEMENT

The authors have no conflict of interest to declare. M. Iemitsu received funding from the Ito Foundation.

## ETHICS STATEMENT

All the participants voluntarily provided written informed consent before participating in the study. This study was approved by the Ethics Committee of Ritsumeikan University (BKC‐2018‐060) and was conducted in accordance with the Declaration of Helsinki. The study was registered in the University Hospital Medical Information Network Clinical Trials Registry (UMIN000038253). Signed informed consent was obtained from all participants.

## Supporting information


Figure S1.

Table S1.

Table S2.

## Data Availability

The datasets generated in this study are available from the corresponding author upon request.
