# Supplementary material for: Effect of resistance training and chicken meat on muscle strength and mass and the gut microbiome of older women: A randomized controlled trial
Source: Physiol Rep. 2024 Jun 18;12(12):e16100. doi: 10.14814/phy2.16100 (PMC11184365; doi:10.14814/phy2.16100)
Supplement: Supplementary file 1 — Figure S1. [file PHY2-12-e16100-s001.pdf]

Supplemental Fig. S1

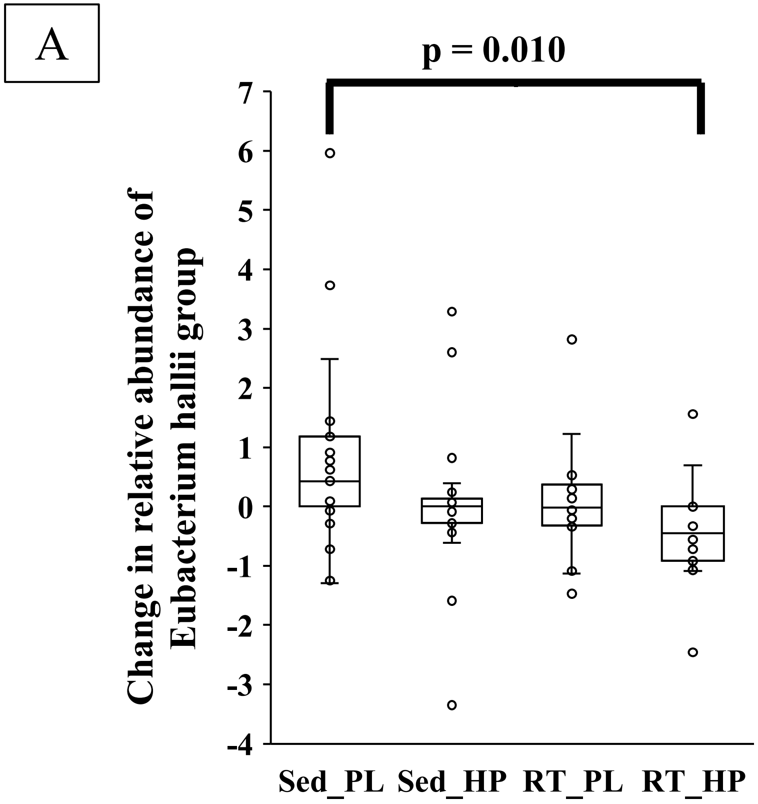

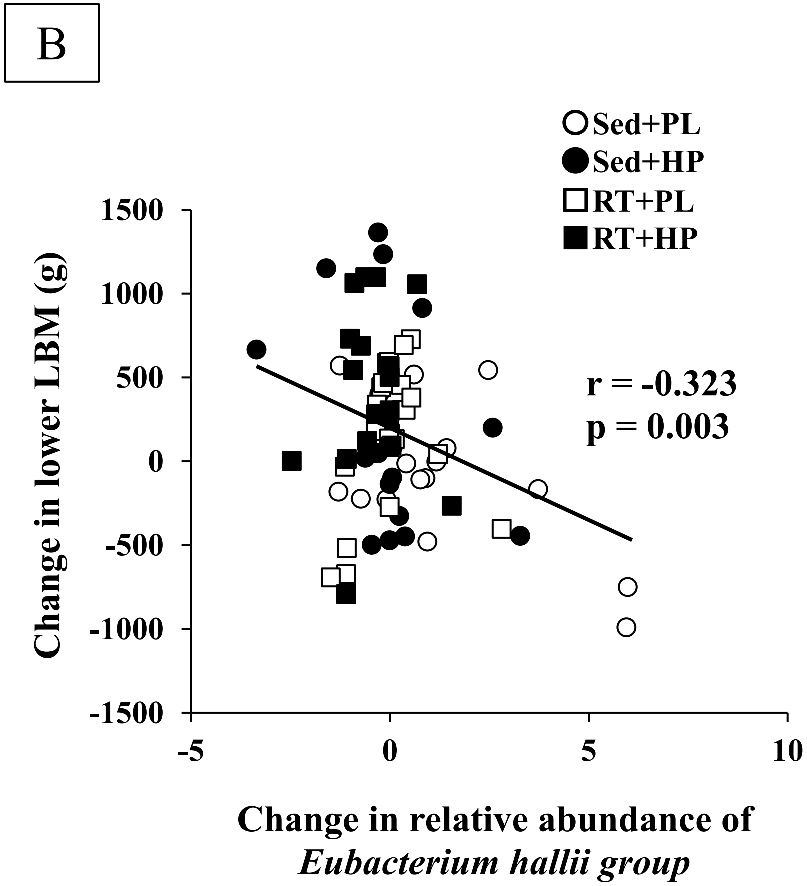

Supplemental Fig. S1. Effects of resistance training and chicken meat intake on the relative abundance of Eubacterium hallii group genera (A) at the genera level among the four groups. Values are expressed as means ± SD. *P < 0.05. Correlation between lower LBM and relative abundance of Eubacterium hallii group genera at the genus level (B).
