# Supplementary material for: Effect of resistance training and chicken meat on muscle strength and mass and the gut microbiome of older women: A randomized controlled trial
Source: Physiol Rep. 2024 Jun 18;12(12):e16100. doi: 10.14814/phy2.16100 (PMC11184365; doi:10.14814/phy2.16100)
Supplement: Supplementary file 2 — Table S1. [file PHY2-12-e16100-s003.docx]

| Supplemental Table S1. Comparison of a-diversity and relative abundance of gut microbes among four group at before and after intervention. | | | | |  |  |  |  |  |  |  |  |  |  |  |  |  |  |  |
| --- | --- | --- | --- | --- | --- | --- | --- | --- | --- | --- | --- | --- | --- | --- | --- | --- | --- | --- | --- |
|  | Sed+PL (N=21) | |  |  |  | Sed+PL (N= 22) | |  |  |  | Sed+PL (N = 20) | |  |  |  | Sed+PL (N = 18) | |  |  |
|  | P-value | FDR | before_mean | after_mean |  | P-value | FDR | before_mean | after_mean |  | P-value | FDR | before_mean | after_mean |  | P-value | FDR | before_mean | after_mean |
| Observed OTUs | 0.001 | 0.009 | 752.238 | 600.095 |  | <0.001 | 0.001 | 718.136 | 511.091 |  | 0.037 | 0.239 | 724.55 | 600.9 |  | <0.001 | 0.002 | 745.5 | 539.222 |
| Chao1 | <0.001 | <0.001 | 1622.172 | 1016.530 |  | <0.001 | 0.002 | 1530.764 | 1061.417 |  | <0.001 | <0.001 | 1574.163 | 1033.103 |  | <0.001 | <0.001 | 1620.031 | 1031.353 |
| Shannon | 0.120 | 0.389 | 4.015 | 3.792 |  | 0.103 | 0.413 | 3.934 | 3.65 |  | 0.883 | 0.979 | 4.048 | 4.037 |  | 0.406 | 0.75 | 3.977 | 3.848 |
| Simpson | 0.382 | 0.612 | 0.930 | 0.903 |  | 0.601 | 0.817 | 0.922 | 0.916 |  | 0.947 | 0.979 | 0.932 | 0.939 |  | 0.913 | 0.913 | 0.925 | 0.932 |
| Fisher | 0.001 | 0.009 | 189.536 | 144.590 |  | <0.001 | 0.001 | 179.315 | 116.577 |  | 0.04 | 0.239 | 180.62 | 144.929 |  | <0.001 | 0.002 | 188.204 | 124.684 |
| D.1..Actinobacteria_D.2..Actinobacteria_D.3..Bifidobacteriales_D.4..Bifidobacteriaceae_D.5..Bifidobacterium | 0.365 | 0.612 | 3.094 | 5.775 |  | 0.159 | 0.477 | 4.448 | 6.88 |  | 0.841 | 0.979 | 3.825 | 4.992 |  | 0.837 | 0.878 | 3.577 | 5.319 |
| D.1..Bacteroidetes_D.2..Bacteroidia_D.3..Bacteroidales_D.4..Bacteroidaceae_D.5..Bacteroides | 0.028 | 0.166 | 31.889 | 21.357 |  | 0.898 | 0.944 | 34.833 | 33.031 |  | 0.398 | 0.956 | 31.952 | 25.872 |  | 0.085 | 0.399 | 39.434 | 28.884 |
| D.1..Bacteroidetes_D.2..Bacteroidia_D.3..Bacteroidales_D.4..Porphyromonadaceae_D.5..Parabacteroides | 0.043 | 0.205 | 2.647 | 2.315 |  | 0.377 | 0.817 | 1.724 | 2.468 |  | 0.776 | 0.979 | 2.758 | 3.266 |  | 0.776 | 0.878 | 3.837 | 3.872 |
| D.1..Bacteroidetes_D.2..Bacteroidia_D.3..Bacteroidales_D.4..Prevotellaceae_D.5..Prevotella 9 | 0.336 | 0.612 | 6.190 | 7.971 |  | 0.141 | 0.477 | 4.694 | 2.509 |  | 0.66 | 0.979 | 2.203 | 3.699 |  | 0.831 | 0.878 | 3.037 | 3.193 |
| D.1..Bacteroidetes_D.2..Bacteroidia_D.3..Bacteroidales_D.4..Prevotellaceae_D.5..Prevotellaceae NK3B31 group | 0.896 | 0.970 | 1.405 | 2.201 |  | 0.610 | 0.817 | 0.973 | 0.695 |  | 0.979 | 0.979 | 1.378 | 1.483 |  | 0.324 | 0.744 | 0.838 | 0.856 |
| D.1..Bacteroidetes_D.2..Bacteroidia_D.3..Bacteroidales_D.4..Rikenellaceae_D.5..Alistipes | 0.534 | 0.754 | 2.384 | 1.086 |  | 0.606 | 0.817 | 2.975 | 2.246 |  | 0.655 | 0.979 | 3.236 | 3.175 |  | 0.282 | 0.744 | 3.062 | 1.928 |
| D.1..Firmicutes_D.2..Clostridia_D.3..Clostridiales_D.4..Lachnospiraceae_D.5...Eubacterium hallii group | 0.285 | 0.588 | 1.256 | 2.255 |  | 0.68 | 0.817 | 1.227 | 1.237 |  | 0.946 | 0.979 | 0.914 | 0.936 |  | 0.356 | 0.744 | 1.366 | 0.941 |
| D.1..Firmicutes_D.2..Clostridia_D.3..Clostridiales_D.4..Lachnospiraceae_D.5...Ruminococcus torques group | 0.99 | 0.99 | 1.683 | 1.701 |  | 0.46 | 0.817 | 1.445 | 1.32 |  | 0.213 | 0.64 | 0.991 | 1.738 |  | 0.815 | 0.878 | 1.326 | 1.601 |
| D.1..Firmicutes_D.2..Clostridia_D.3..Clostridiales_D.4..Lachnospiraceae_D.5..Anaerostipes | 0.92 | 0.97 | 1.286 | 1.111 |  | 0.438 | 0.817 | 1.347 | 0.755 |  | 0.745 | 0.979 | 1.234 | 1.381 |  | 0.296 | 0.744 | 1.629 | 2.776 |
| D.1..Firmicutes_D.2..Clostridia_D.3..Clostridiales_D.4..Lachnospiraceae_D.5..Blautia | 0.056 | 0.224 | 4.119 | 7.474 |  | 0.002 | 0.01 | 3.596 | 8.367 |  | 0.021 | 0.239 | 2.308 | 3.645 |  | 0.327 | 0.744 | 3.665 | 6.623 |
| D.1..Firmicutes_D.2..Clostridia_D.3..Clostridiales_D.4..Lachnospiraceae_D.5..Fusicatenibacter | 0.138 | 0.389 | 1.041 | 1.57 |  | 0.944 | 0.944 | 0.635 | 1.465 |  | 0.457 | 0.979 | 1.246 | 1.53 |  | 0.1 | 0.399 | 0.889 | 1.959 |
| D.1..Firmicutes_D.2..Clostridia_D.3..Clostridiales_D.4..Lachnospiraceae_D.5..Lachnoclostridium | 0.279 | 0.588 | 1.62 | 2.467 |  | 0.734 | 0.838 | 1.69 | 1.844 |  | 0.12 | 0.575 | 1.25 | 1.397 |  | 0.812 | 0.878 | 2.803 | 2.336 |
| D.1..Firmicutes_D.2..Clostridia_D.3..Clostridiales_D.4..Lachnospiraceae_D.5..Lachnospira | 0.294 | 0.588 | 1.112 | 0.726 |  | 0.078 | 0.375 | 1.847 | 0.638 |  | 0.763 | 0.979 | 1.226 | 1.027 |  | 0.372 | 0.744 | 1.55 | 0.731 |
| D.1..Firmicutes_D.2..Clostridia_D.3..Clostridiales_D.4..Lachnospiraceae_D.5..Roseburia | 0.146 | 0.389 | 2.469 | 3.534 |  | 0.534 | 0.817 | 1.779 | 2.682 |  | 0.304 | 0.811 | 1.677 | 2.817 |  | 0.055 | 0.329 | 1.954 | 3.285 |
| D.1..Firmicutes_D.2..Clostridia_D.3..Clostridiales_D.4..Ruminococcaceae_D.5..Faecalibacterium | 0.93 | 0.97 | 5.062 | 5.574 |  | 0.681 | 0.817 | 4.107 | 4.819 |  | 0.168 | 0.637 | 7.464 | 6.366 |  | 0.837 | 0.878 | 5.78 | 7.089 |
| D.1..Firmicutes_D.2..Clostridia_D.3..Clostridiales_D.4..Ruminococcaceae_D.5..Subdoligranulum | 0.753 | 0.951 | 1.59 | 1.217 |  | 0.548 | 0.817 | 2.242 | 1.734 |  | 0.871 | 0.979 | 2.953 | 2.779 |  | 0.776 | 0.878 | 1.613 | 2.193 |
| D.1..Firmicutes_D.2..Negativicutes_D.3..Selenomonadales_D.4..Acidaminococcaceae_D.5..Phascolarctobacterium | 0.534 | 0.754 | 1.294 | 0.964 |  | 0.618 | 0.817 | 1.507 | 1.225 |  | 0.491 | 0.979 | 1.585 | 1.314 |  | 0.701 | 0.878 | 1.649 | 1.22 |
| D.1..Proteobacteria_D.2..Betaproteobacteria_D.3..Burkholderiales_D.4..Alcaligenaceae_D.5..Sutterella | 0.927 | 0.97 | 0.842 | 0.987 |  | 0.911 | 0.944 | 0.726 | 0.928 |  | 0.935 | 0.979 | 1.064 | 1.015 |  | 0.841 | 0.878 | 1.307 | 0.995 |
| D.1..Proteobacteria_D.2..Gammaproteobacteria_D.3..Enterobacteriales_D.4..Enterobacteriaceae_D.5..Escherichia Shigella | 0.715 | 0.951 | 3.703 | 2.798 |  | 0.487 | 0.817 | 1.91 | 1.66 |  | 0.186 | 0.637 | 2.808 | 1.684 |  | 0.46 | 0.789 | 0.347 | 0.637 |
| The values are expressed as mean ± SD. Sed+PL: sedentary-control with placebo, Sed+HP: sedentary-control with chicken meat, RT+PL: resistance training with placebo, RT+HP: resistance training with chicken meat. | | | | | | | | | | | | | | | | | | | |
