# Supplementary material for: Effect of resistance training and chicken meat on muscle strength and mass and the gut microbiome of older women: A randomized controlled trial
Source: Physiol Rep. 2024 Jun 18;12(12):e16100. doi: 10.14814/phy2.16100 (PMC11184365; doi:10.14814/phy2.16100)
Supplement: Supplementary file 3 — Table S2. [file PHY2-12-e16100-s002.docx]

| Supplemental Table S2. Comparison of changes in a-diversity and relative abundance of gut microbes before and after interventions in Sed+HP, RT+PL, or RT+HP group with Sed+PL group | | | | |  |
| --- | --- | --- | --- | --- | --- |
| Sed+HP |  |  |  |  |  |
|  | p-value | FDR | Sed+PL_change_mean | Sed+HP_change_mean |  |
| Observed OTUs | 0.504 | 0.893 | −152.143 | −207.045 |  |
| Chao1 | 0.444 | 0.865 | −605.642 | −469.347 |  |
| Shannon | 0.585 | 0.906 | −0.223 | −0.284 |  |
| Simpson | 0.362 | 0.865 | −0.026 | −0.005 |  |
| Fisher | 0.536 | 0.893 | −44.945 | −62.738 |  |
| D.1..Actinobacteria_D.2..Actinobacteria_D.3..Bifidobacteriales_D.4..Bifidobacteriaceae_D.5..Bifidobacterium | 0.688 | 0.906 | 2.681 | 2.432 |  |
| D.1..Actinobacteria_D.2..Coriobacteriia_D.3..Coriobacteriales_D.4..Coriobacteriaceae_D.5..Collinsella | 0.971 | 0.971 | −0.074 | 0.190 |  |
| D.1..Bacteroidetes_D.2..Bacteroidia_D.3..Bacteroidales_D.4..Bacteroidaceae_D.5..Bacteroides | 0.070 | 0.433 | −10.531 | −1.802 |  |
| D.1..Bacteroidetes_D.2..Bacteroidia_D.3..Bacteroidales_D.4..Porphyromonadaceae_D.5..Parabacteroides | 0.001** | 0.029 | −0.331 | 0.745 |  |
| D.1..Bacteroidetes_D.2..Bacteroidia_D.3..Bacteroidales_D.4..Prevotellaceae_D.5..Prevotella.9 | 0.051 | 0.433 | 1.781 | −2.185 |  |
| D.1..Bacteroidetes_D.2..Bacteroidia_D.3..Bacteroidales_D.4..Prevotellaceae_D.5..Prevotellaceae.NK3B31.group | 0.957 | 0.971 | 0.796 | −0.279 |  |
| D.1..Bacteroidetes_D.2..Bacteroidia_D.3..Bacteroidales_D.4..Rikenellaceae_D.5..Alistipes | 0.903 | 0.971 | −1.298 | −0.728 |  |
| D.1..Firmicutes_D.2..Clostridia_D.3..Clostridiales_D.4..Lachnospiraceae_D.5...Eubacterium..hallii.group | 0.053 | 0.433 | 0.999 | 0.010 |  |
| D.1..Firmicutes_D.2..Clostridia_D.3..Clostridiales_D.4..Lachnospiraceae_D.5...Ruminococcus..torques.group | 0.253 | 0.718 | 0.018 | −0.125 |  |
| D.1..Firmicutes_D.2..Clostridia_D.3..Clostridiales_D.4..Lachnospiraceae_D.5..Anaerostipes | 0.409 | 0.865 | −0.174 | −0.592 |  |
| D.1..Firmicutes_D.2..Clostridia_D.3..Clostridiales_D.4..Lachnospiraceae_D.5..Blautia | 0.206 | 0.718 | 3.355 | 4.771 |  |
| D.1..Firmicutes_D.2..Clostridia_D.3..Clostridiales_D.4..Lachnospiraceae_D.5..Fusicatenibacter | 0.662 | 0.906 | 0.529 | 0.830 |  |
| D.1..Firmicutes_D.2..Clostridia_D.3..Clostridiales_D.4..Lachnospiraceae_D.5..Lachnoclostridium | 0.259 | 0.718 | 0.847 | 0.155 |  |
| D.1..Firmicutes_D.2..Clostridia_D.3..Clostridiales_D.4..Lachnospiraceae_D.5..Lachnospira | 0.087 | 0.433 | −0.386 | −1.209 |  |
| D.1..Firmicutes_D.2..Clostridia_D.3..Clostridiales_D.4..Lachnospiraceae_D.5..Roseburia | 0.159 | 0.662 | 1.065 | 0.903 |  |
| D.1..Firmicutes_D.2..Clostridia_D.3..Clostridiales_D.4..Ruminococcaceae_D.5..Faecalibacterium | 0.618 | 0.906 | 0.512 | 0.712 |  |
| D.1..Firmicutes_D.2..Clostridia_D.3..Clostridiales_D.4..Ruminococcaceae_D.5..Subdoligranulum | 0.770 | 0.906 | −0.374 | −0.508 |  |
| D.1..Firmicutes_D.2..Negativicutes_D.3..Selenomonadales_D.4..Acidaminococcaceae_D.5..Phascolarctobacterium | 0.797 | 0.906 | −0.330 | −0.281 |  |
| D.1..Proteobacteria_D.2..Betaproteobacteria_D.3..Burkholderiales_D.4..Alcaligenaceae_D.5..Sutterella | 0.790 | 0.906 | 0.144 | 0.201 |  |
| D.1..Proteobacteria_D.2..Gammaproteobacteria_D.3..Enterobacteriales_D.4..Enterobacteriaceae_D.5..Escherichia.Shigella | 0.450 | 0.865 | −0.905 | −0.250 |  |
|  |  |  |  |  |  |
| RT+PL |  |  |  |  |  |
|  | p-value | FDR | Sed+PL_change_mean | Sed+HP_change_mean |  |
| Observed OTUs | 0.557 | 0.872 | −152.143 | −123.650 |  |
| Chao1 | 0.907 | 0.907 | −605.642 | −541.060 |  |
| Shannon | 0.206 | 0.872 | −0.223 | −0.011 |  |
| Simpson | 0.115 | 0.872 | −0.026 | 0.007 |  |
| Fisher | 0.523 | 0.872 | −44.945 | −35.691 |  |
| D.1..Actinobacteria_D.2..Actinobacteria_D.3..Bifidobacteriales_D.4..Bifidobacteriaceae_D.5..Bifidobacterium | 0.744 | 0.907 | 2.681 | 1.168 |  |
| D.1..Actinobacteria_D.2..Coriobacteriia_D.3..Coriobacteriales_D.4..Coriobacteriaceae_D.5..Collinsella | 0.804 | 0.907 | −0.074 | 0.177 |  |
| D.1..Bacteroidetes_D.2..Bacteroidia_D.3..Bacteroidales_D.4..Bacteroidaceae_D.5..Bacteroides | 0.506 | 0.872 | −10.531 | −6.081 |  |
| D.1..Bacteroidetes_D.2..Bacteroidia_D.3..Bacteroidales_D.4..Porphyromonadaceae_D.5..Parabacteroides | 0.027* | 0.686 | −0.331 | 0.508 |  |
| D.1..Bacteroidetes_D.2..Bacteroidia_D.3..Bacteroidales_D.4..Prevotellaceae_D.5..Prevotella.9 | 0.437 | 0.872 | 1.781 | 1.497 |  |
| D.1..Bacteroidetes_D.2..Bacteroidia_D.3..Bacteroidales_D.4..Prevotellaceae_D.5..Prevotellaceae.NK3B31.group | 0.719 | 0.907 | 0.796 | 0.105 |  |
| D.1..Bacteroidetes_D.2..Bacteroidia_D.3..Bacteroidales_D.4..Rikenellaceae_D.5..Alistipes | 0.583 | 0.872 | −1.298 | −0.061 |  |
| D.1..Firmicutes_D.2..Clostridia_D.3..Clostridiales_D.4..Lachnospiraceae_D.5...Eubacterium..hallii.group | 0.060 | 0.753 | 0.999 | 0.022 |  |
| D.1..Firmicutes_D.2..Clostridia_D.3..Clostridiales_D.4..Lachnospiraceae_D.5...Ruminococcus..torques.group | 0.334 | 0.872 | 0.018 | 0.747 |  |
| D.1..Firmicutes_D.2..Clostridia_D.3..Clostridiales_D.4..Lachnospiraceae_D.5..Anaerostipes | 0.335 | 0.872 | −0.174 | 0.147 |  |
| D.1..Firmicutes_D.2..Clostridia_D.3..Clostridiales_D.4..Lachnospiraceae_D.5..Blautia | 0.348 | 0.872 | 3.355 | 1.337 |  |
| D.1..Firmicutes_D.2..Clostridia_D.3..Clostridiales_D.4..Lachnospiraceae_D.5..Fusicatenibacter | 0.419 | 0.872 | 0.529 | 0.284 |  |
| D.1..Firmicutes_D.2..Clostridia_D.3..Clostridiales_D.4..Lachnospiraceae_D.5..Lachnoclostridium | 0.593 | 0.872 | 0.847 | 0.147 |  |
| D.1..Firmicutes_D.2..Clostridia_D.3..Clostridiales_D.4..Lachnospiraceae_D.5..Lachnospira | 0.489 | 0.872 | −0.386 | −0.199 |  |
| D.1..Firmicutes_D.2..Clostridia_D.3..Clostridiales_D.4..Lachnospiraceae_D.5..Roseburia | 0.886 | 0.907 | 1.065 | 1.141 |  |
| D.1..Firmicutes_D.2..Clostridia_D.3..Clostridiales_D.4..Ruminococcaceae_D.5..Faecalibacterium | 0.426 | 0.872 | 0.512 | −1.098 |  |
| D.1..Firmicutes_D.2..Clostridia_D.3..Clostridiales_D.4..Ruminococcaceae_D.5..Subdoligranulum | 0.814 | 0.907 | −0.374 | −0.175 |  |
| D.1..Firmicutes_D.2..Negativicutes_D.3..Selenomonadales_D.4..Acidaminococcaceae_D.5..Phascolarctobacterium | 0.885 | 0.907 | −0.330 | −0.271 |  |
| D.1..Proteobacteria_D.2..Betaproteobacteria_D.3..Burkholderiales_D.4..Alcaligenaceae_D.5..Sutterella | 0.770 | 0.907 | 0.144 | −0.049 |  |
| D.1..Proteobacteria_D.2..Gammaproteobacteria_D.3..Enterobacteriales_D.4..Enterobacteriaceae_D.5..Escherichia.Shigella | 0.229 | 0.872 | −0.905 | −1.124 |  |
|  |  |  |  |  |  |
| RT+HP |  |  |  |  |  |
|  | p-value | FDR | Sed+PL_change_mean | Sed+HP_change_mean |  |
| Observed OTUs | 0.662 | 0.905 | −152.143 | −206.278 |  |
| Chao1 | 0.989 | 1.000 | −605.642 | −588.678 |  |
| Shannon | 0.602 | 0.905 | −0.223 | −0.128 |  |
| Simpson | 0.172 | 0.716 | −0.026 | 0.007 |  |
| Fisher | 0.642 | 0.905 | −44.945 | −63.520 |  |
| D.1..Actinobacteria_D.2..Actinobacteria_D.3..Bifidobacteriales_D.4..Bifidobacteriaceae_D.5..Bifidobacterium | 0.291 | 0.807 | 2.681 | 1.743 |  |
| D.1..Actinobacteria_D.2..Coriobacteriia_D.3..Coriobacteriales_D.4..Coriobacteriaceae_D.5..Collinsella | 0.238 | 0.793 | −0.074 | −0.097 |  |
| D.1..Bacteroidetes_D.2..Bacteroidia_D.3..Bacteroidales_D.4..Bacteroidaceae_D.5..Bacteroides | 0.811 | 0.905 | −10.531 | −10.549 |  |
| D.1..Bacteroidetes_D.2..Bacteroidia_D.3..Bacteroidales_D.4..Porphyromonadaceae_D.5..Parabacteroides | 0.147 | 0.716 | −0.331 | 0.036 |  |
| D.1..Bacteroidetes_D.2..Bacteroidia_D.3..Bacteroidales_D.4..Prevotellaceae_D.5..Prevotella.9 | 1.000 | 1.000 | 1.781 | 0.156 |  |
| D.1..Bacteroidetes_D.2..Bacteroidia_D.3..Bacteroidales_D.4..Prevotellaceae_D.5..Prevotellaceae.NK3B31.group | 0.380 | 0.905 | 0.796 | 0.018 |  |
| D.1..Bacteroidetes_D.2..Bacteroidia_D.3..Bacteroidales_D.4..Rikenellaceae_D.5..Alistipes | 0.472 | 0.905 | −1.298 | −1.134 |  |
| D.1..Firmicutes_D.2..Clostridia_D.3..Clostridiales_D.4..Lachnospiraceae_D.5...Eubacterium..hallii.group | 0.005** | 0.136 | 0.999 | −0.425 |  |
| D.1..Firmicutes_D.2..Clostridia_D.3..Clostridiales_D.4..Lachnospiraceae_D.5...Ruminococcus..torques.group | 0.833 | 0.905 | 0.018 | 0.276 |  |
| D.1..Firmicutes_D.2..Clostridia_D.3..Clostridiales_D.4..Lachnospiraceae_D.5..Anaerostipes | 0.019* | 0.233 | −0.174 | 1.147 |  |
| D.1..Firmicutes_D.2..Clostridia_D.3..Clostridiales_D.4..Lachnospiraceae_D.5..Blautia | 0.439 | 0.905 | 3.355 | 2.958 |  |
| D.1..Firmicutes_D.2..Clostridia_D.3..Clostridiales_D.4..Lachnospiraceae_D.5..Fusicatenibacter | 0.254 | 0.793 | 0.529 | 1.070 |  |
| D.1..Firmicutes_D.2..Clostridia_D.3..Clostridiales_D.4..Lachnospiraceae_D.5..Lachnoclostridium | 0.047* | 0.392 | 0.847 | −0.468 |  |
| D.1..Firmicutes_D.2..Clostridia_D.3..Clostridiales_D.4..Lachnospiraceae_D.5..Lachnospira | 0.767 | 0.905 | −0.386 | −0.819 |  |
| D.1..Firmicutes_D.2..Clostridia_D.3..Clostridiales_D.4..Lachnospiraceae_D.5..Roseburia | 0.833 | 0.905 | 1.065 | 1.331 |  |
| D.1..Firmicutes_D.2..Clostridia_D.3..Clostridiales_D.4..Ruminococcaceae_D.5..Faecalibacterium | 0.563 | 0.905 | 0.512 | 1.309 |  |
| D.1..Firmicutes_D.2..Clostridia_D.3..Clostridiales_D.4..Ruminococcaceae_D.5..Subdoligranulum | 0.102 | 0.635 | −0.374 | 0.581 |  |
| D.1..Firmicutes_D.2..Negativicutes_D.3..Selenomonadales_D.4..Acidaminococcaceae_D.5..Phascolarctobacterium | 0.722 | 0.905 | −0.330 | −0.429 |  |
| D.1..Proteobacteria_D.2..Betaproteobacteria_D.3..Burkholderiales_D.4..Alcaligenaceae_D.5..Sutterella | 0.746 | 0.905 | 0.144 | −0.312 |  |
| D.1..Proteobacteria_D.2..Gammaproteobacteria_D.3..Enterobacteriales_D.4..Enterobacteriaceae_D.5..Escherichia.Shigella | 0.525 | 0.905 | −0.905 | 0.290 |  |
| The values are expressed as mean ± SD. Sed+PL: sedentary-control with placebo, Sed+HP: sedentary-control with chicken meat, RT+PL: resistance training with placebo, RT+HP: resistance training with chicken meat, . *P < 0.05 vs. Sed+PL, **P < 0.01 vs. Sed+PL. | | | | |  |
|  |  |  |  |  |  |
